# Boosting of the enhanced permeability and retention effect with nanocapsules improves the therapeutic effects of cetuximab

**DOI:** 10.20892/j.issn.2095-3941.2019.0292

**Published:** 2020-05-15

**Authors:** Chao Yang, Yanli Tan, Hongzhao Qi, Junhu Zhou, Lixia Long, Qi Zhan, Yunfei Wang, Xubo Yuan, Chunsheng Kang

**Affiliations:** ^1^Department of Neurosurgery, Tianjin Medical University General Hospital, Tianjin 300052, China; ^2^Tianjin Neurological Institute, Key Laboratory of Post-neurotrauma Neuro-repair and Regeneration in Central Nervous System, Ministry of Education and Tianjin City, Tianjin 300052, China; ^3^Department of Pathology, Affiliated Hospital of Hebei University, Baoding 071000, China; ^4^Department of Pathology, Hebei University Medical College, Baoding 071000, China; ^5^Tianjin Key Laboratory of Composite and Functional Materials, School of Material Science and Engineering, Tianjin University, Tianjin 300072, China; ^6^Institute for Translational Medicine, Qingdao University, Qingdao 266021, China; ^7^Affiliated Cancer Hospital & Institute of Guangzhou Medical University, Guangzhou 510095, China

**Keywords:** Endothelial cells, EPR effect, nanocapsule, single dose administration, therapeutic antibody

## Abstract

**Objective:** The introduction of therapeutic antibodies (tAbs) into clinical practice has revolutionized tumor treatment strategies, but their tumor therapy efficiency is still far below expectations because of the rapid degradation and limited tumor accumulation of tAbs.

**Methods**: We developed a nanocapsule-based delivery system to induce the self-augmentation of the enhanced permeability and retention (EPR) effect. This system constantly penetrated across the blood-tumor barrier into the tumor while avoiding the attack of tAbs by the immune system. The biodistribution and therapeutic effect were tested with single dose administration of nanocapsule-tAbs *in vivo*.

**Results**: The accumulation of Nano(cetuximab) within subcutaneous PC9 tumors was gradually enhanced over 6 days after single dose administration, which was contrary to the biodistribution of native cetuximab. Nano(cetuximab) accumulated in tumor tissues *via* the EPR effect and released cetuximab. The released cetuximab acted on vascular endothelial cells to destroy the blood-tumor barrier and induce self-augmentation of the EPR effect, which in turn contributed to further tumor accumulation of long-circulating Nano(cetuximab). Compared with single dose administration of native cetuximab, Nano(cetuximab) showed an effective tumor suppressive effect for 3 weeks.

**Conclusions**: The nanocapsule-based delivery system efficiently delivered tAbs to tumor tissues and released them to boost the EPR effect, which facilitated further tumor accumulation of the tAbs. This novel self-augmentation of the EPR effect facilitated by the biological characteristics of tAbs and nanotechnology contributed to the improvement of the therapeutic effect of tAbs, and stimulated new ideas for antibody-based tumor therapy.

## Introduction

Therapeutic antibodies (tAbs), which can specifically target a wide variety of receptors expressed on the surface of cancer cells to induce apoptosis, are currently a standard component of tumor therapy^1–6^. The introduction of these tAbs into clinical practice revolutionized treatment strategies for several solid tumors, but the rapid degradation and limited tumor accumulation of tAbs have made them a suboptimal choice for treatment of such tumors^7,8^. To further overcome the above limitations, researchers have used nanoparticles, such as silica-based nanoparticles, peptide- or protein-based nanoparticles, and polymeric nanoparticles, as vectors for tAb delivery^9–12^. These nanoparticles avoid attack of tAbs by enzymes and immune cells, efficiently maintaining the integrity of tAb structure and function during circulation^13^. However, this strategy does not improve the tumor accumulation ability of tAbs because the penetration of macromolecular drugs (molecule weights > 40 kDa) and nanoparticles into tumor tissues depends on the enhanced permeability and retention (EPR) effect^14,15^. Therefore, an ideal tAb delivery system should protect the tAbs and simultaneously enhance their tumor accumulation.

Augmentation of the EPR effect has been used to enhance the tumor accumulation of macromolecular drugs or nanoparticles. Exogenous stimulating factors, such as angiotensin and nitric oxide (NO)-releasing compounds, induce systemic hypertension or increased blood flow^16^. Under these conditions, macromolecular drugs or nanoparticles are released into the interstitial space or matrix of tumor tissues by hydrodynamic forces^17^. The combined application of exogenous stimulating factors and nanotechnology could be a theoretical choice for tAb delivery. However, the potential side effects of exogenous stimulating factors may limit their clinical application. For example, NO may promote tumor growth^18^. Many tAbs bind to vascular endothelial cells within tumors, such as endothelial cells in several neoplasms, which have been shown to express the EGFR, and bind to cetuximab^19^. These tAbs block receptors to induce apoptosis in endothelial cells, increasing vascular permeability^20^. We therefore hypothesized that tAbs possess the ability to self-augment the EPR effect. Accordingly, we used nanocapsules, which encapsulated tAbs in their core to protect the tAbs from degradation by enzymes and immune cells. These nanocapsules showed a tumor microenvironment-responsive degradation characteristic, and were quickly degraded in the tumor to release the tAbs. These released tAbs then acted on vascular endothelial cells in the tumor to induce self-augmentation of the EPR effect, which further enhanced the tumor accumulation of circulating nanocapsules.

Taking advantage of nanotechnology and the physiological properties of tAbs, we simultaneously protected tAbs and enhanced their tumor accumulation, dramatically improving their therapeutic effects. Electron microscopy revealed moderate to severe vascular endothelial injury after 6 days of Nano(cetuximab) administration, while mild injury to the endothelium was observed in native cetuximab-treated mice. Single dose administration of Nano(cetuximab) therefore continuously destroyed the vascular endothelium to boost the EPR effect, which in turn contributed to the accumulation of Nano(cetuximab) penetrating across the blood-tumor barrier. The nanocapsule-based delivery system increased the EPR effect and penetrated the blood-tumor barrier, which will further improve our understanding of the EPR effect. The therapeutic effects of a therapeutic antibody delivered by nanocapsules in single dose administration will therefore potentially benefit patients with epithelial cancers.

## Materials and methods

### Synthesis of monoclonal antibody (mAb) nanocapsules

A cetuximab (ERBITUX, Merck, Darmstadt, Germany) solution (5 mg/mL) was diluted to 1 mg/mL using phosphate buffer (20 mM, pH = 7.4) in an ice bath. N-(3-aminopropyl) methacrylamide (APM) was added to the protein solution with stirring for 10 min at 4 °C. Then, 2-methacryloyloxyethyl phosphorylcholine (MPC) monomers^13,21–23^ and diallyl disulfide (DADS) were added sequentially to the protein solution with stirring. The MPC:APM:crosslinker molar ratio was adjusted to 50:5:1. Radical polymerization was initiated by adding both ammonium persulfate and N,N,N´,N´-tetramethylethylenediamine (TEMED) to the reaction solution. The polymerization was allowed to proceed for 2 h in a nitrogen atmosphere at 4 °C. The synthesized nanocapsules were purified by passing through a hydrophobic interaction column (Phenyl-Sepharose 4BCL; GE Healthcare, Sunnyvale, CA, USA) and dialyzed against phosphate-buffered saline (PBS).

The protocol for the synthesis of fluorescein-labeled nanocapsules was similar to the above procedure. To label mAbs with fluorescein, the mAbs and fluorescein [fluorescein isothiocyanate (FITC) or NHS-Cy5.5] were mixed (molar ratio was 1:1) in 100 mM PBS (pH = 8.0) and reacted for 2 h, and then the unreacted fluorescein was removed by dialyzing against PBS (20 mM, pH = 7.4).

### Cell culture

The RAW 264.7 and PC9 cell lines (mouse macrophages) were purchased from the American Type Culture Collection (Manassas, VA, USA) and cultured in Dulbecco’s Modified Eagle’s Medium (DMEM; Gibco, Gaithersburg, MD, USA). The experimental cells were cultured in DMEM supplemented with 10% fetal bovine serum (Gibco), 100 units of penicillin/mL (Sigma-Aldrich, St. Louis, MO, USA), and 100 μg/mL streptomycin (Sigma-Aldrich). The cells were grown at 37 °C in an incubator with 5% CO_2_. PC9 cells were cultured in 6-well plates for 24 h and then treated with different groups of drugs for 24 h. The cells were rinsed with PBS three times to remove the culture media, followed by RIPA lysis buffer containing protease inhibitors (phenylmethylsulfonyl fluoride; Gibco) and phosphatase inhibitors (Gibco) for total protein collection.

### Western blot

Protein was extracted from cells that were seeded into 6-well plates at a density of 2 × 10^5^ cells/well and cultured for attachment, and Nano(cetuximab) was pretreated with dithiothreitol (DTT) for 1 h at 37 °C. Native cetuximab or DTT-treated Nano(cetuximab) was added to the wells (0.1 mg/mL) for 48 h. The cells were then homogenized in RIPA lysis buffer (Solarbio, Beijing, China) containing a Protease Inhibitor Cocktail (Beyotime, Beijing, China) and phosphatase inhibitor cocktail (Beyotime). Tissue lysates were incubated on ice for 30 min and centrifuged at 12,000 × *g* at 4 °C for 20 min. The supernatant was collected, and protein concentrations were measured by a Coomassie Blue staining kit (Beyotime). Equal amounts of total protein per lane were separated using 5% and 10% SDS-PAGE and then transferred to a polyvinylidene difluoride membrane. The membranes were incubated with primary antibodies against EGFR (1:1000; Cell Signaling Technology, Danvers, MA, USA), p-EGFR (1:1000; Cell Signaling Technology), Akt (1:1000; Cell Signaling Technology), p-Akt (1:1000; Cell Signaling Technology), and glyceraldehyde 3-phosphate dehydrogenase (1:1000; Cell Signaling Technology) overnight at 4 °C, followed by incubation with horseradish peroxidase-conjugated anti-rabbit or anti-mouse secondary antibodies. The protein signals were detected with the G:BOX F3 imaging system (Syngene, Cambridge, UK).

### The nude mouse subcutaneous tumor model

BALB/c-A nude mice (female, 4-weeks-old, 13–15 g) were purchased from the Animal Center at the Cancer Institute at the Chinese Academy of Medical Science (Beijing, China). The animal use protocol was reviewed and approved by the Animal Ethical and Welfare Committee (TMUaMEC 2018034). A total of 5 × 10^5^ PC9 cells were implanted to establish a subcutaneous tumor model. Cetuximab (a total of 100 μg for each mouse, intravenous injection) and Nano(cetuximab) (a total of 100 μg for each mouse, intravenous injection) labeled with Cy5.5 were injected only once during the experimental period. All mouse experiments were performed according to protocols approved by the institutional animal care and use committee.

### Transmission electron microscopy (TEM) of nanocapsules and tumor vessels

Five μL of nanocapsule (0.1 mg/mL) was dropped onto carbon-coated copper grids. After 5 min, excess amounts of samples were removed. The grid was then rinsed and stained with 2% (w/v) phosphotungstic acid solution.

The mouse models with the PC9 tumor cell xenografts were injected with control reagents, antibodies, and nanocapsules through the tail vein on the 10th day after implantation, and then sacrificed on the 1st and 6th days. The subcutaneous tumor xenograft was removed, rapidly cut into small particles of approximately 1 mm^3^, and immersed in 2.5% glutaraldehyde (w/v) for 2 h. After washing with PBS (0.01 M, pH 7.4), the sample solution was stained with 2% (v/v) tannic acid and 1% OsO_4_ solution, then dehydrated with ethanol solution, transferred to isoamyl acetate, and dried in liquid CO_2_ at the critical point. The endothelial cells of the two groups of tumor tissues were observed using a JEM-2100Plus TEM microscope (JEOL, Tokyo, Japan).

### Hematoxylin and eosin (H&E) staining, immunohistochemistry (IHC), and confocal imaging

Paraffin-embedded tissue sections were used for H&E staining. For IHC analysis, sections were incubated with primary antibodies [1:100 dilution; anti-p-EGFR and anti-p-AKT antibodies were purchased from Cell Signaling Technology (CST), and an anti-Ki67 antibody was purchased from Zsgb Bio (Beijing, China)] overnight at 4 °C, followed by a 1 h incubation at 37 °C with a biotinylated secondary antibody (1:100 dilution). The samples were then incubated with horseradish peroxidase labeled streptomycoidin and DAB (diaminobenzidine), counterstained with hematoxylin, and visualized using a light microscope. RAW 264.7 cells were seeded on 10% collagen-coated glass coverslips and then incubated with cetuximab or nanocapsules for 0, 1, 2 and 4 h. Actin protein was stained by phalloidin. The EGFR protein was labeled with antibodies against EGFR (1:100; CST). Primary antibody labeling was detected by Alexa Fluor 488-conjugated secondary antibodies (Proteintech, Rosemount, IL, USA), followed by confocal imaging with an Olympus FluoView 1200 system (Olympus, Tokyo, Japan). All confocal scanning parameters were held constant among the samples, and the images were minimally processed to maintain the integrity of the data.

### Statistical analysis

Statistical analyses were performed using GraphPad Prism 6.0 (GraphPad, La Jolla, CA, USA). Data are expressed as the mean ± SD and analyzed by one-way analysis of variance for multiple comparisons or Student’s *t*-test (two-tailed) for comparing two groups. Statistical significance was set at a value of *P* < 0.05.

## Results

### Synthesis and characterization of MPC nanocapsules

**Figure 1:**
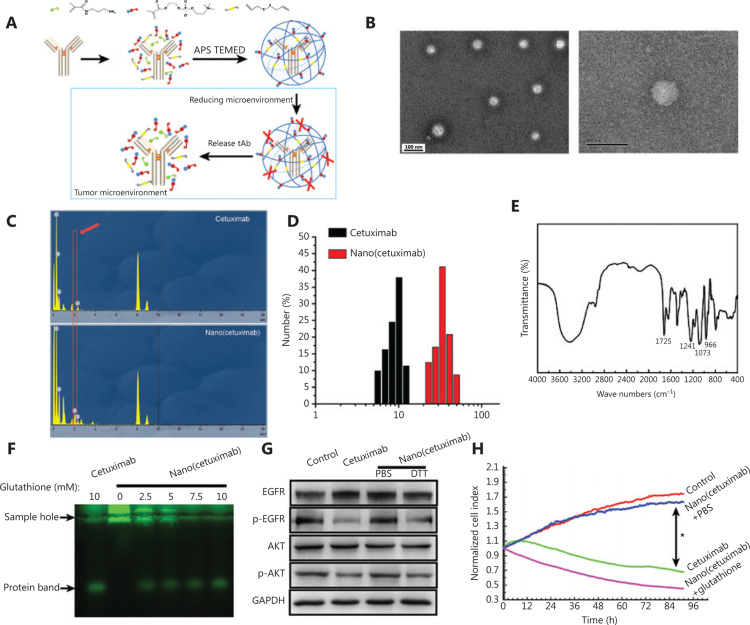
(A) The preparation of therapeutic antibody tAb nanocapsules. (B) A representative transmission electron microscopy image of Nano(cetuximab). Scale bar: 100 μm. (C) The results of energy spectrum analysis of native cetuximab and Nano(cetuximab). (D) The size distributions of cetuximab and Nano(cetuximab). (E) Fluorescein isothiocyanate results of Nano(cetuximab). (F) Agarose gel electrophoresis results of Nano(cetuximab) after treatment with glutathione and (G) Western blot analysis of the activity of native cetuximab and Nano(cetuximab). (H) RTCA (Real Time Cellular Analysis) showing the cell proliferation profile after incubation with various groups.

The preparation of Nano(cetuximab) is shown in **Figure 1A**. APM and MPC were used as monomers, and DADS was used as the crosslinker. These monomers and crosslinkers aggregated around cetuximab at 4 °C, relying on electrostatic interactions and hydrogen bond interactions. Ammonium persulfate (APS) and TEMED triggered the polymerization of the monomers and crosslinkers to form Nano(cetuximab)* in situ*. The tAbs-encapsulation ratio was 20%–30%.

To verify the formation of Nano(cetuximab), the morphologies of Nano(cetuximab) were observed by TEMA. Representative TEM images showed that the morphologies of Nano(cetuximab) were regular and spherical, with a uniform diameter of 30 ± 5 nm (**Figure 1B**). The energy spectrum analysis showed that phosphorus existed in a sample of Nano(cetuximab) (**Figure 1C**). The results further proved that natural cetuximab did not contain phosphorus, so the particles containing cross-linking agent or monomer were generated after *in situ* polymerization. Dynamic light scattering indicated that the mean diameter of Nano(cetuximab) was ˜32 nm (**Figure 1D**), which was consistent with the TEM results. The Fourier transform infrared spectroscopy spectrum of the nanocapsules exhibited new peaks at 1725, 1241, 1073, and 966 cm^−1^ (**Figure 1F**), which were characteristic peaks of MPC-based polymer networks (**Figure 1E**)^24^.

The responsive-release ability of Nano(cetuximab) was tested by agarose gel electrophoresis (**Figure 1F**) and SDS-PAGE (**Supplementary Figure S1**). Agarose gel electrophoresis results showed that glutathione-treated nanocapsules showed the same FITC fluorescence bands as the original antibody group. In view of the uniformity of the Nano(cetuximab) particles, one single protein band was observed on the SDS-PAGE gel. DTT was used as the reducing agent to degrade disulfide bonds. Compared with untreated Nano(cetuximab), the predominant bands of DTT-treated Nano(cetuximab) and cetuximab were both at 50 kD to 175 kD, indicating that Nano(cetuximab) could be degraded under reducing conditions. In addition, the predominant bands of DTT-treated cetuximab and Nano(cetuximab) were both at 50 kD, further supporting this conclusion.

In addition, Western blot analysis was used to investigate the activity of native cetuximab and Nano(cetuximab) (**Figure 1G**). Cetuximab bound to EGFR to inhibit its phosphorylation and block the AKT signaling pathway, downregulating the activated forms of EGFR (p-EGFR) and AKT (p-AKT). As expected, PC9 cells treated with DTT-treated Nano(cetuximab) or native cetuximab had similar levels of p-EGFR and p-AKT, further confirming the activity of the released cetuximab. To verify the proliferative inhibitory activity of Nano(cetuximab), we conducted *in vitro* biosafety and cancer treatment experiments based on cell lines. **Figure 1H** shows that when PC9 cells were incubated with bovine serum albumin, Nano(cetuximab) showed similar viabilities, which were significantly higher than those with native cetuximab and Nano(cetuximab) (treated with glutathione). 

### Novel nanocapsules prolonged the circulation time of cetuximab mAbs and delivered antibodies to the tumor microenvironment

**Figure 2:**
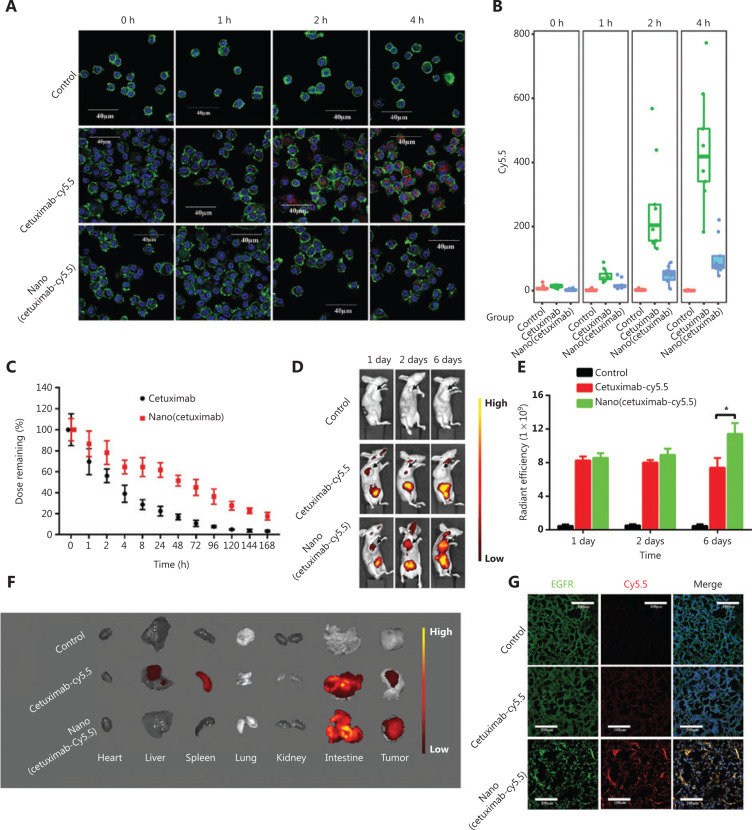
(A) Cy5.5-labeled cetuximab incubated with macrophages for 0, 1, 2 and 4 h and imaged by confocal scanning microscopy; green represents actin, and red represents cetuximab, Scale bar: 40 μm. (B) Quantitation of the fluorescence intensity of the uptake of native cetuximab and Nano(cetuximab) by macrophages. (C) Blood clearance profiles of native cetuximab and Nano(cetuximab) in healthy female Kunming mice. (D) Representative fluorescence images of tumor-bearing mice at 1, 2, and 6 days after treatment with Cy5.5-labeled native cetuximab or Nano(cetuximab). (E) Radiant efficiencies of Cy5.5-labeled native cetuximab and Nano(cetuximab) in the subcutaneous tumor model. (F) Representative *ex vivo* fluorescence images of major organs. (G) Accumulation of Cy5.5-labeled native cetuximab and Nano(cetuximab) in tumor sections evaluated using a fluorescence microscope; green represents ERG, and red represents cetuximab. Scale bar: 100 μm. The significance level is shown as ^*^P < 0.01.

The elemental composition of the polymer shell was MPC, which endowed the polymer shell with antiprotein adsorption and immune system escape. The uptake efficiencies of native cetuximab and Nano(cetuximab) by macrophages were evaluated (**Figure 2A**). Cetuximab labeled with Cy5.5 was incubated with macrophages, and confocal microscopy showed that the Cy5.5 signal in the macrophages gradually increased. In contrast, the Cy5.5 signal of Nano(cetuximab) was unobservable even at 4 h. The quantitation of fluorescence intensity further confirmed the above conclusions (**Figure 2B**). The *in vivo* circulation time of cetuximab and Nano(cetuximab) was also tested (**Figure 2C**). The results showed that the half-life of native cetuximab was less than 4 h, which was much less than that of Nano(cetuximab). The half-life of Nano(cetuximab) was ˜48 h, and approximately 20% of the administered dose of Nano(cetuximab) remained in the blood circulation even after 1 week. These results indicated that the nanocapsules could efficiently protect cetuximab.

The *in vivo* distributions of native cetuximab and Nano(cetuximab) were evaluated. Cetuximab and Nano(cetuximab), both labeled with Cy5.5, were injected into nude mice implanted with subcutaneous lung cancer cells. **Figure 2D** shows the *in vivo* bioluminescence images of tumor-bearing mice. The fluorescence signal of native cetuximab indicated that cetuximab accumulated in the tumor site at 1 day after injection. However, over time, the tumor accumulation of cetuximab gradually decreased. In contrast, the accumulation of Nano(cetuximab) in the tumor site gradually increased during the observation period. Semiquantitative analysis showed that the fluorescent signal intensity of Nano(cetuximab) at 6 days was approximately 1.5 times that of native cetuximab (**Figure 2E**). On the sixth day, we performed Cy5.5 fluorescence detection on different organs. Consistent with the *in vivo* imaging results, the fluorescent signals of tumors in the Nano(cetuximab) group were significantly higher than that of the tumors in the native cetuximab group (**Figure 2F**). **Figure 2G** shows confocal scanning microscopy images of tumor sections. The nanocapsules dramatically improved the tumor accumulation of cetuximab, which bound with EGFR.

### Damaged tumor vessels boosted the EPR effect

**Figure 3:**
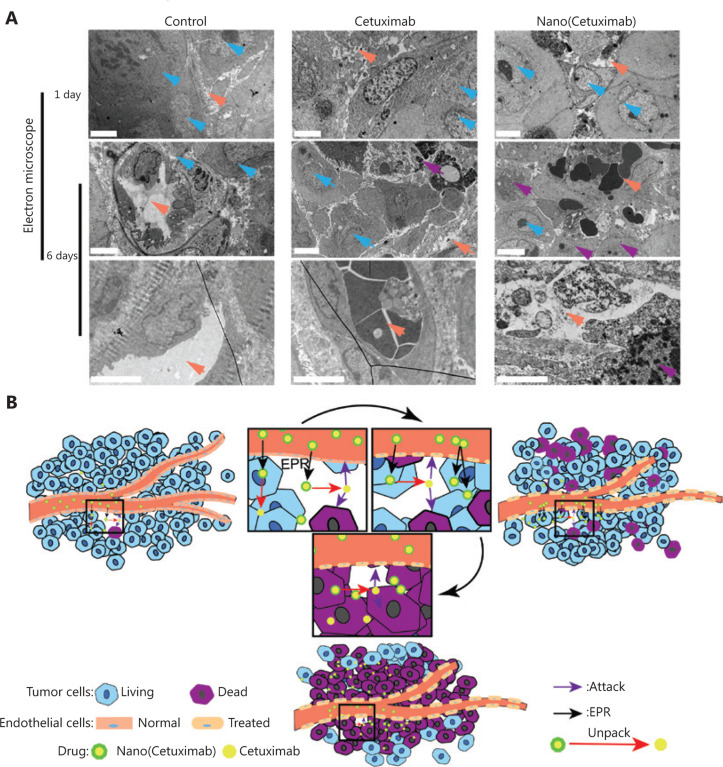
(A) Electron microscopy analysis of the vasculature of tumors at 1 and 6 days after the administration of native cetuximab and Nano(cetuximab). Orange represents the vessel lumen, blue represents tumor cells, and purple represents apoptotic tumor cells. Scale bar: 4 μm. (B) A potential schematic illustration of the working mechanism of Nano(cetuximab).

To characterize the unconventional *in vivo* distribution of Nano(cetuximab), the morphologies of blood vessels in tumor tissues were observed. **Figure 3A** shows electron microscopy results, and the orange arrow indicates the lumen of a blood vessel. The results showed that tumor vascular integrity was not significantly different between the cetuximab and Nano(cetuximab) groups at 1 day after injection. However, at 6 days after injection, the tumor vascular integrity in the Nano(cetuximab) group was lower than that in the cetuximab group (**Figure 3A**). In addition, the number of apoptotic tumor cells, which are indicated by purple arrows, in the Nano(cetuximab) group, was much higher than that in the cetuximab group. These results were consistent with our expectations. **Figure 3B** shows a possible mechanism of how Nano(cetuximab) accumulates in tumor tissues *via* the EPR effect in the initial phase. In addition, the nanoparticles were degraded in the tumor microenvironment to release native cetuximab, which could act on tumor cells to induce apoptosis. More importantly, the released cetuximab also bound EGFR expressed on vascular endothelial cells, to damage the vasculature of the tumor. Due to the long circulation time of Nano(cetuximab), the tumor vasculature in the Nano(cetuximab) group was continuously attacked by antibodies, and the vascular permeability was significantly increased. At the same time, the long-circulating Nano(cetuximab) further passed through the tumor blood vessels to accumulate in the tumor tissues, inducing apoptosis in more tumor cells. This self-augmentation of the EPR effect was the collective result of the targeting ability of tAbs and the long-circulating property endowed by the nanocapsules. 

### Reduced side effects

**Figure 4:**
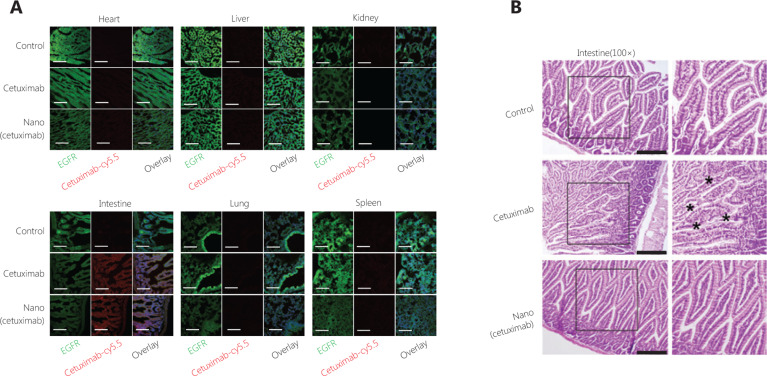
(A) Confocal scanning microscopy images of frozen sections. Green represents EGFR, and red represents cetuximab. Scale bar: 100 μm. (B) Hematoxylin and eosin staining of the intestine. Scale bar: 400 μm.

To further investigate the organ distribution of Nano(cetuximab), confocal scanning of the major organs was performed. **Figure 4A** shows that the fluorescence signal was highest in the intestines, which was consistent with the *ex vivo* fluorescence images of the major organs. The results of H&E staining of the intestines showed that high accumulation of native cetuximab resulted in damage to the intestines (**Figure 4B**). In contrast, H&E staining of the intestines in Nano(cetuximab)-treated mice indicated that Nano(cetuximab) did not cause side effects on the 6th day. These results may have resulted from the tumor microenvironment-responsive release characteristics. Together, the results showed that nanocapsules efficiently reduced the potential side effects of tAbs. 

### Chemotherapeutic effects of Nano(cetuximab) *in vivo*

**Figure 5:**
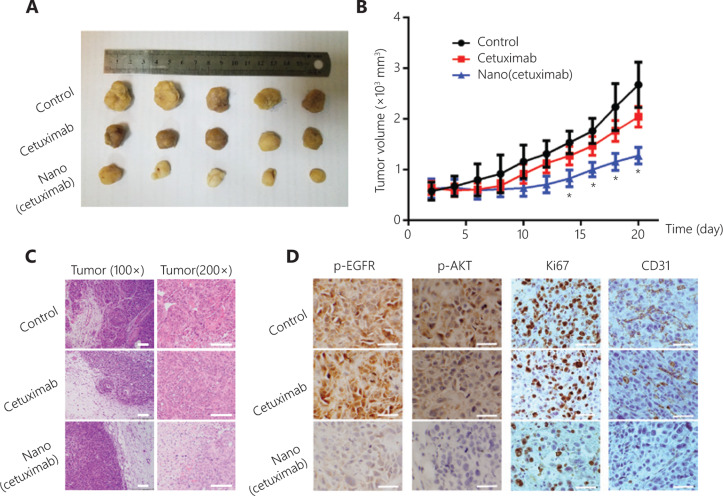
(A) Photographs of tumors after treatment on day 21. (B) The tumor volume was measured every 2 days. The significance levels are shown as ^*^P < 0.01. (C) Representative photomicrographs showing hematoxylin and eosin staining. Scale bar: 200 μm. (D) Immunohistochemical staining for p-EGFR, p-AKT, Ki67, and CD31 in tissue samples. Scale bar: 50 μm.

The *in vivo* tumor suppressive effect of Nano(cetuximab) was tested. Semiquantitative analysis of the tumor weights and volumes in three groups of mice showed that the tumor weights were not significantly different among the three groups, but that the volume was significantly lower in the Nano(cetuximab) group than in the cetuximab and control groups after only one injection (**Figure 5A, B**). H&E staining of subcutaneous lung cancer tissues from mice indicated that many small solid tumors appeared around the tumor boundary in the control and cetuximab groups, but were absent in the Nano(cetuximab) group (**Figure 5C**). Because EGFR signaling is known to regulate cell proliferation and angiogenesis in many cancers^25–27^, IHC analysis was conducted and the results showed that Nano(cetuximab) treatment specifically downregulated p-EGFR and p-AKT expression, and that the expression of the cell proliferation biomarker Ki67 was also reduced. The expression of p-EGFR and p-AKT in tumors was significantly lower for mice treated with Nano(cetuximab), which was approximately 30% of those treated with native cetuximab (**Figure 5D**). In a similar manner, the percentages of proliferation marker Ki67 positive cells in the tumor tissue were also reduced from about 35%–80% with Nano(cetuximab) treatment. IHC staining for CD31, a biomarker of angiogenesis, showed a significant decrease (**Figure 5D**). Together, these results indicated that tAbs retained their biofunction after being released from nanocapsules in the tumor microenvironment, and that a single dose of Nano(cetuximab) was more effective than a single dose of cetuximab alone.

## Discussion

Therapeutic antibody-based treatment of cancer has been established during the last 30 years as one of the most successful therapeutic strategies for both hematological malignancies and solid tumors^28,29^. During the process of tAb development, the tumor therapy efficiency gradually increases, but it is still far from the expected efficiency^30^. The tumor therapy efficiency of tAbs mainly depends on their tumor accumulation efficiency, because the targets of the tAbs are on the surface of tumor cells^17,20,31–34^. To further enhance the therapeutic efficiency of tAbs, their tumor accumulation must therefore be enhanced. Extending the retention of tAbs in the blood is a feasible way, and this is also the goal pursued by current delivery system. For example, tAbs have evolved from being fully murine, to chimeric, to humanized, and to fully human antibodies to reduce the immunogenicity of tAbs. In addition, excipients, such as Tween and sucrose, have been used to increase the circulation time of tAbs^35^. In actual practice, the amount of tAbs that penetrates the blood-tumor barrier rather than the amount retained in the blood directly determines the tumor accumulation of tAbs. To date, however, the blood-tumor barrier penetration efficiency of tAbs has been rarely characterized^36,37^.

As typical macromolecular drugs, tAbs can break through the blood-tumor barrier and accumulate in tumor sites due to the “EPR” effect^15,37–39^. Augmentation of the EPR effect can therefore be helpful for the blood-tumor barrier penetration capability of tAbs. In the present study, we utilized the characteristics of tAbs to induce their self-augmentation of the EPR effect. We showed that tAbs bound to receptors expressed on tumor vascular endothelial cells to induce cell apoptosis. The apoptosis of endothelial cells leads to increased vascular permeability, which results in augmentation of the EPR effect (**Figure 3**). The results of *in vivo* distribution indicated that more tAbs accumulated at the tumor site because of augmentation of the EPR effect (**Figures 2 and 5**). To the best of our knowledge, this is the first report to characterize the effect of tAbs on the EPR effect, which could stimulate new ideas for antibody-based tumor therapy.

It should be especially noted that the self-augmentation of the EPR effect by tAbs builds on their extended blood circulation time. The destruction of the blood-tumor barrier requires sufficient tAbs to act on endothelial cells for a long period of time. For example, 1 day after injection, the vascular destruction in the cetuximab and Nano(cetuximab) groups showed no significant difference (**Figure 3A**). In contrast, at 6 days after injection, vascular destruction in the Nano(cetuximab) group was more pronounced (**Figure 3A**). However, tAbs should exist in the blood after destruction of the blood-tumor barrier to enhance the amount that accumulates in the tumor (**Figures 2 and 3**). This is the reason why the tumor accumulation of Nano(cetuximab) gradually increased.

To improve the circulation time of tAbs, we constructed nanocapsules (**Figure 1**). These nanocapsules protected tAbs from immune clearance, and the half-life of cetuximab was extended from ˜4 h to ˜48 h (**Figure 2C**). Furthermore, approximately 20% of the injected Nano(cetuximab) still existed in the blood circulation even after 1 week (**Figure 2C**). It should be noted that the cetuximab used in this research was purified by dialysis. Generally, the circulation time of injected cetuximab in clinical patients is similar to that of Nano(cetuximab), because excipients have been added to the clinical injection. Clinical injections of tAbs may have the self-augmentation phenomenon of the EPR effect. Despite this possibility, we are still optimistic about nanocapsule-based tAbs because of their *in vivo* stability conferred by the covalent polymer shell and the tumor responsive-release characteristics.

## Conclusions

We constructed nanocapsules to efficiently deliver and release tAbs into tumor tissues. These released tAbs acted on vascular endothelial cells and disrupted the blood tumor barrier, resulting in increased accumulation of tAbs at the tumor site. This novel self-augmentation of the EPR effect induced by the biological characteristics of tAbs and nanotechnology properties contributed to the improvement of therapeutic effects of tAbs, and could stimulate new ideas for treatment.
