# Supplementary material for: Boosting of the enhanced permeability and retention effect with nanocapsules improves the therapeutic effects of cetuximab
Source: Cancer Biol Med. 2020 May 15;17(2):433–43. doi: 10.20892/j.issn.2095-3941.2019.0292 (PMC7309461; doi:10.20892/j.issn.2095-3941.2019.0292)
Supplement: Supplementary file 1 [file cbm-17-433-s001.pdf]

Supplementary material

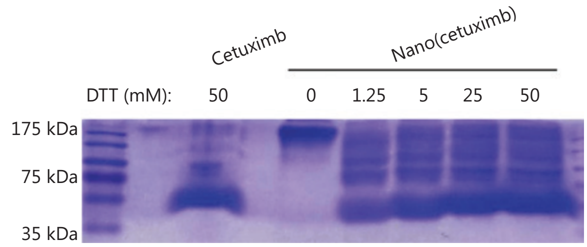

Figure S1 Nano(cetuximab) was treated with dithiothreitol, and Coomassie Brilliant Blue staining was conducted after reducing polyacrylamide gel electrophoresis.
